# Pollution Sources and Carcinogenic Risk of PAHs in PM_1_ Particle Fraction in an Urban Area

**DOI:** 10.3390/ijerph17249587

**Published:** 2020-12-21

**Authors:** Ivana Jakovljević, Zdravka Sever Štrukil, Ranka Godec, Ivan Bešlić, Silvije Davila, Mario Lovrić, Gordana Pehnec

**Affiliations:** 1 Institute for Medical Research and Occupational Health, Ksaverska cesta 2, 10000 Zagreb, Croatia; zsever@imi.hr (Z.S.Š.); rgodec@imi.hr (R.G.); ibeslic@imi.hr (I.B.); sdavila@imi.hr (S.D.); gpehnec@imi.hr (G.P.); 2 Know-Center, Inffeldgasse 13, 8010 Graz, Austria; mlovric@know-center.at

**Keywords:** BaP, HPLC, carcinogenic, diagnostic ratio

## Abstract

Airborne particles are composed of inorganic species and organic compounds. PM_1_ particles, with an aerodynamic diameter smaller than 1 μm, are considered to be important in the context of adverse health effects. Many compounds bound to particulate matter, such as polycyclic aromatic hydrocarbons (PAH), are suspected to be genotoxic, mutagenic, and carcinogenic. In this study, PAHs in the PM_1_ particle fraction were measured for one year (1/1/2018–31/12/2018). The measuring station was located in the northern residential part of Zagreb, the Croatian capital, close to a street with modest traffic. Significant differences were found between PAH concentrations during cold (January–March, October–December) and warm (April–September) periods of the year. In general, the mass concentrations of PAHs characteristic for car exhausts (benzo(ghi)perylene (BghiP), indeno(1,2,3-cd)pyrene (IP), and benzo(b)fluoranthene (BbF)) were higher during the whole year than concentrations of fluoranthene (Flu) and pyrene (Pyr), which originated mostly from domestic heating and biomass burning. Combustion of diesel and gasoline from vehicles was found to be one of the main PAH sources. The incremental lifetime cancer risk (ILCR) was estimated for three age groups of populations and the results were much lower than the acceptable risk level (1 × 10^−6^). However, more than ten times higher PAH concentrations in the cold part of the year, as well as associated health risk, emphasize the need for monitoring of PAHs in PM_1_. These data represent a valuable tool in future plans and actions to control PAH sources and to improve the quality of life of urban populations.

## 1. Introduction

Particulate matter (PM) is assumed to be among the most hazardous of all ambient pollutants. Particle pollution contains “inhalable coarse particles” with diameters larger than 2.5 μm and smaller than 10 μm and “fine particles” with diameters of 2.5 μm or smaller. One of the most significant organic groups bound to PM in terms of health risk are polycyclic aromatic hydrocarbons (PAH). These compounds can exist in the atmosphere in the vapor phase (PAHs with low molecular weight), whereas heavier ones (PAHs with high molecular weight) are mostly adsorbed on the particle phase [1]. PAHs generally occur as complex mixtures, products of incomplete combustion processes, and originate from natural and anthropogenic sources [2]. High PAH levels in the ambient air of large metropolitan cities are usually linked with traffic, as well as diesel and gasoline automobiles [1,3]. Catalytic converters have shown a significant effect on reducing levels of the PAH concentrations in exhaust gases, but PAH emission levels continue to increase due to the contribution of other sources, such as traffic congestion [4]. PAHs are always emitted as a mixture, and the molecular concentration ratios are considered to be typical for a given emission source [5]. The toxicity, carcinogenicity, and mutagenicity of aromatic hydrocarbons have led to increased concerns for human populations. Long-term exposure to PAHs can cause toxic effects such as breathing problems, lung function abnormalities, decreased immune function, kidney and liver damage, skin irritation, and inflammation. The most significant health effect to be expected from inhalation of PAHs is increased risk of lung cancer primarily in occupations with heavy exposure to traffic-related air pollution, such as policeman [6] and newsagents [7]. Piccardo et al. [8] noticed that taxi drivers in Genoa were exposed to significantly higher daily BaP concentrations in comparison to workers from another occupational category. Fuel and biomass combustions, traffic emissions, and use of lubricant oils were identified as the main sources of PAH exposures at five Portuguese fire stations. During a normal shift in non-fire situations, levels of light PAHs were predominant and may promote some adverse health outcomes [9]. Bladder cancer is also linked to exposure to PAHs. Boada et al. [10] conducted a case study in the Canary Islands measuring PAH serum levels of 140 patients diagnosed for bladder cancer. Their results showed a difference in PAH contamination profile between patients and the control group, leading to the conclusion that specific PAH mixtures play a role in bladder cancer. People are persistently exposed to PAHs. Benzo(a)pyrene (BaP) is the most investigated PAH, and most information on the toxicity and manifestation of PAHs is related to this compound, which is why it was used as an indicator of carcinogenic hazard in polluted environments [11,12,13].

Although particle composition has been determined by many authors, there is still not much research related to the study of PAHs in airborne fine particles. Fine particles, with an aerodynamic diameter of less than 2.5 μm (PM_2.5_), and especially particles with aerodynamic diameter of less than 1 μm (PM_1_), may play an important role in affecting human health. Squizzato et al. [14] stated the following reasons: they penetrate more effectively into the deep lung; they can penetrate more readily into indoor environments; they can remain suspended for longer periods of time in the atmosphere than coarse particles; they may be transported over long distances; they tend to carry higher concentrations of more toxic compounds, including acids, heavy metals, and organic compounds; and they have a larger surface area per unit mass compared to larger particles and can thus absorb larger amounts of semi-volatile compounds. Measurements of PM_1_ and its content have so far not been included in routine measurement programs, although they would provide more information about potential PM sources and could be used to improve PM control strategies and health protection. A recent study by Yang et al. [15] found that both PM_1_ and PM_2.5_ levels were associated with poorer lung function in children, with stronger associations for PM_1_ compared to PM_2.5_, pointing to the importance of regulating finer PM fractions. Furthermore, a lot of previous health risk estimations were carried out taking into account larger fractions of particulate matter (PM_10_ or even total suspended particles), which do not enter deeply into the respiratory system [16,17,18].

Studies regarding PAH in PM_1_ were carried out only at a few urban locations in Europe [19,20,21,22,23,24,25]. It was found that the levels varied significantly between heating and non-heating season [22,23,26]. The highest concentrations were observed during winter in areas where coal and wood burning were used for heating [22,24]. Traffic-loaded sites showed a large contribution of PAHs with larger molecular weight [21,22,27,28].

In this study, the mass concentrations of individual PAHs in the PM_1_ particle fraction were determined in an urban area together with some gaseous pollutants. The relationship between measured species and meteorological parameters (temperature, relative humidity, atmospheric pressure, wind direction, and velocity) was determined. Potential pollution sources were assessed using PAH diagnostic ratios, Spearman’s regression, and principal component analysis (PCA). In a previous study at the same location [19], factor analysis (FA) was used to identify pollution sources. The aim of both PCA and FA is to determine the main relationships between the observed variables and to reduce the large number of observed variables to a smaller number of factors. Mathematically, in the PCA, the entire variance in the observed variables is analyzed, while in the FA only the mutual variance is analyzed. Attempts are made to identify and eliminate variance due to error and variance specific to each individual variable. The assumption of FA is that factors “create” variable values, while in PCA it is assumed that variables “create” components. In PCA, there is no basic theory that would define how variables are grouped into factors, the variables are simply empirically related within components. As atmospheric PAH levels are affected by different weather conditions and numerous atmospheric reactions, and it is not possible to determine their interdependencies for each day, we estimated that the PCA method is more applicable for this analysis. In addition, the adverse health effect of PAHs on a human organism was assessed using toxic equivalent concentrations. The incremental lifetime cancer risk (ILCR) was estimated for three age group of populations, which presents the first such study carried out for PAHs in PM_1_ in this part of Europe.

## 2. Materials and Methods

### 2.1. Location and Sampling

Concentrations of 11 PAHs in PM_1_ particle fraction were measured continuously from January to December 2018, together with gaseous pollutants (CO, NO_2_, SO_2_, and O_3_). The measuring station (45°50′6.83″ N, 15°58′42.12″ E, 168 m a.s.l.) was situated in the northern residential area of Zagreb, the Croatian capital (~800.000 inhabitants). It is a low-rise building area with small inhabitant density and mild traffic density. Residential (domestic) heating relies mostly on gas, but some households still use oil or wood for heating and cooking.

Samples of PM_1_ particle fraction were collected on quartz filters with low-volume sequential automatic sampler (Sven Leckel) from about 55 m^3^ of air per day. Filters were collected 24-h a day, and the total number of samples was 363. The sampler inlet was located approximately 1.5 m above ground and 15 m away from the road. The PM_1_ samples were kept frozen in aluminum foil at −18 °C until PAH analysis to avoid PAH losses and sample degradation. The filters were extracted no later than two months after sampling.

### 2.2. Measurements of Gaseous Pollutants and Meteorological Data

Gaseous pollutants were measured using automatic devices. SO_2_ was determined using a HORIBA APSA 370 device according to the norm (EN 1421:2014). NO_2_ was measured by a HORIBA APNA 370 device (according the norm EN 14211:2012), O_3_ was measured using a HORIBA APOA 370 device (according the norm EN 14625:2012), and CO was measured using a HORIBA APMA 370 device (according the norm (EN 14626:2012). Measurements of SO_2_, CO, NO_2_, and O_3_ are part of the local air quality monitoring network funded by the City of Zagreb, City Office for Economy, Energetics and Environment Protection.

Meteorological data (temperature, relative humidity, pressure, amount of rainfall, wind direction, and velocity) were obtained from the nearest meteorological station (Maksimir) of the Croatan Hydrological and Meteorological Service (www.meteo.hr).

### 2.3. Analysis of PAHs

Extraction of PAHs from filters was performed with a solvent mixture (toluene:cyclohexane 7:3) in an ultrasonic bath. Further preparation included centrifugation (10 min, 3000 rpm) and evaporation to dryness. After that, samples were redissolved in acetonitrile. Concentrations of PAHs were determined by Agilent Infinity 1260 high-performance liquid chromatography (HPLC) with a fluorescence detector. Zorbax Eclipse PAH column (100 × 4.6 mm) was used for separations of PAHs. The mobile phase was acetonitrile and water (60:40), with a flow rate of 1 mL min^−1^ [29,30]. Calibration curves were prepared with a commercial PAH standard (Supelco EPA 610 PAHs Mix). The calibration range was from 0.005 ng μL^−1^ to 0.08 ng μL^−1^ for pyrene, benzo(a)anthracene, chrysene, benzo(k)fluoranthene, benzo(a)pyrene, and indeno(1,2,3,cd)pyrene, but for fluoranthene, benzo(j)fluoranthene, benzo(b)fluoranthene, dibenzo(ah)anthracene, and benzo(ghi)perilene the calibration range was from 0.01 ng μL^−1^ to 0.16 ng μL^−1^. Samples were analyzed for the following PAHs: fluoranthene (Flu), pyrene (Pyr), benzo(a)anthracene (BaA), chrysene (Chry), benzo(j)fluoranthene (BjF), benzo(b)fluoranthene (BbF), benzo(k)fluoranthene (BkF), benzo(a)pyrene (BaP), dibenzo(ah)anthracene (DahA), benzo(ghi)perylene (BghiP), and indeno(1,2,3-cd)pyrene (IP). The limit of detection (LoD) was from 0.001 ng m^−3^ for BaA to 0.03 ng m^−3^ for BjF. The quantification limit (QL) varied from 0.002 ng m^−3^ for BaA to 0.1 ng m^3^ for BjF. The accuracy of the method was determined by analyzing the standard reference material (SRM 1649a, urban dust) provided by the National Institute of Standards and Technology (NIST) and ranged from 88% for Flu to 109% for BkF. Samples of SRM 1649a were processed the same way as real samples. The details of detection and quantification limits, as well as accuracy are shown in Table S1 of the Supplementary Data section.

### 2.4. Statistical Analysis

The statistical results were processed by Microsoft Excel and the Statistica 13 (Tibco Software Inc.) program. Statistical significance was set at 5% (*p* < 0.05). The Shapiro-Wilk test was used to test the normality of variables. Seasonal differences between warm and cold period for each PAH concentrations, heavy and light PAHs, and PM_1_ particles were tested by Mann–Whitney U test.

The concentration ratios between selected PAHs were investigated to find out more about the nature of pollution sources. Diagnostic ratios are common tools for the identification of pollution sources [30,31,32,33]. However, some papers have presented their restrictions. Katsoyiannis et al. [34] demonstrated that diagnostic ratios cannot be effective as markers of sources, because they are influenced by weather condition and atmospheric reaction of PAHs such as photodegradation, and reaction with ozone and other atmospheric pollutants. To minimize confounding factors such as dissimilarities in volatility, water solubility, and adsorption, diagnostic ratio calculations usually are restricted to PAHs of similar molecular mass [35]. Due to the restrictions of the diagnostic ratio, the multivariate principal component analysis (PCA) was performed on data and calculated anomalies for the studied samples. Principal components (PCs) with eigenvalues greater than 1 for both periods were calculated, and their contributions in the total variance were determined. We graphically displayed the PCs loading as well as the orientation of the variables and samples with respect to these principal components.

### 2.5. Carcinogenic Activity and Population Exposure

Benzo(a)pyrene was used as an indicator of health impact of the PAH mixture to human health. Many studies have shown that BaP was present at more than 50% in total carcinogenic activity and that made it a good indicator of carcinogenic activity of the PAH mixture. The carcinogenic activity of individual PAHs was estimated on the basis of toxic equivalency factors (TEFs) from the literature. Different TEF schemes are developed by different authors, based on experiments in animals [36,37,38,39]. Nisbet and LaGoy [39] completed a new list of TEFs, which appears to better reflect the current state of knowledge on the relative potency of individual PAHs. In this study we used Nisbet and LaGoy’s [39] toxic equivalent factors.

For calculating the risk of the PAH mixture in ambient air, the carcinogenic potencies of individual PAHs are expressed relative to the potency of BaP. BaP equivalents (BaP_eq_) were calculated by multiplying the mass concentration of an individual PAH with its respective toxic equivalency factor. Total carcinogenic potency (TCP) was calculated by summing up the BaP_eq_ of each measured PAH. The equation used to calculate the TCP is presented below (1):

TCP = ƩBaP_eq_ = Ʃγi·TEFi (1)

TCP—total carcinogenic potency/ng m^−3^
BaP_eq_—BaP equivalents concentration
TEFi—toxic equivalency factor of a particular PAH
γi—mass concentration of individual PAH/ng m^−3.^

To determine the daily population exposures, total carcinogenic potency was used to calculate the daily dose according to Equation (3). In this study we tried to estimate the most probable scenario for three age groups: infant (0–1 year), children (5–19 year), and adult (20–70 year). If we assumed that people spent an average of ten hours at their job/school and eight to ten hours at home (including sleeping), we can assume that they were elsewhere for the rest of their day, due to the fact that people spend approximately 25% of their time outdoors, that is 6 h per day.

The incremental lifetime cancer risk (ILCR) posed by exposure to PM_1_-bounded PAHs was computed following Equation (2) [20].

ILCR = ((SF_inh_ × IEL × EF × ED)/(BW × AT × cf)) × y (2)

SF_inh_—inhalation cancer slope factor of BaP/kg day mg^−1^
IEL—BaP_eq_ daily dose/ng day^−1^
EF—exposure frequency/day year^−1^
ED—daily exposure level/μg g^−1^
BW—body weight/kg
AT—average time/day
Cf—conversion factor (10^−6^)
y—age

In this study, SFinh, EF, and ED was used as derived by Chen and Liao [17]. Parameters were different for infants, children, and for adults, with average body weights of 6.79 kg, 36.24 kg, and 59.78 kg, respectively. Parameters that are used in calculation of ILCR are shown in Table S3 of the Supplementary Data section. Results of ILCR shows incremental cancer risk per year and for lifetime cancer risk this result should be multiplied by the age of the person.

In this study, a SFinh of 3.14 kg day mg^−1^ was used, and the proper description of how this value was derived was explained by Chen and Liao [17]. The BaP_eq_ daily dose was calculated by multiplying the TCP concentrations (ng m^−3^), inhalation rate (IR, m^3^ day^−1^), and daily exposure time span (t, h). The equation used to calculate the IEL is presented below (3):

IEL = TCP × t × IR(3)

## 3. Results and Discussion

### 3.1. PAH Concentrations

**Figure 1:**
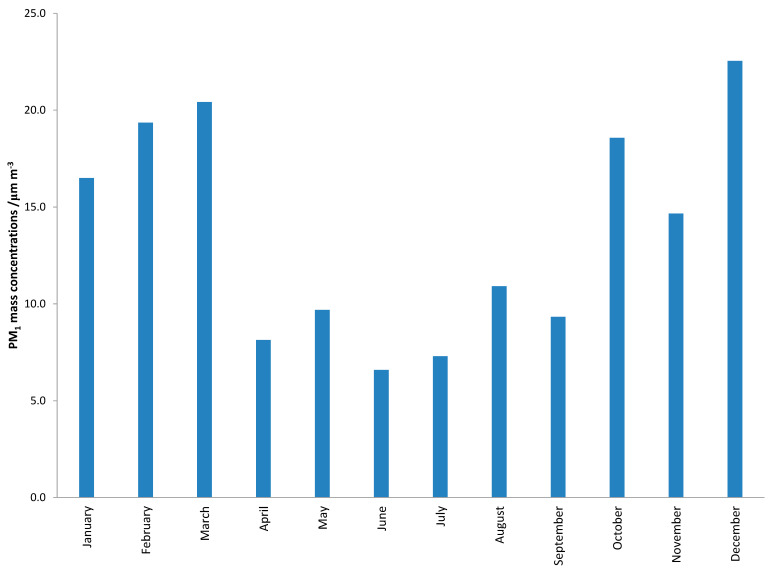
Monthly mass concentrations of the PM_1_ particle fraction during one calendar year.

**Figure 2:**
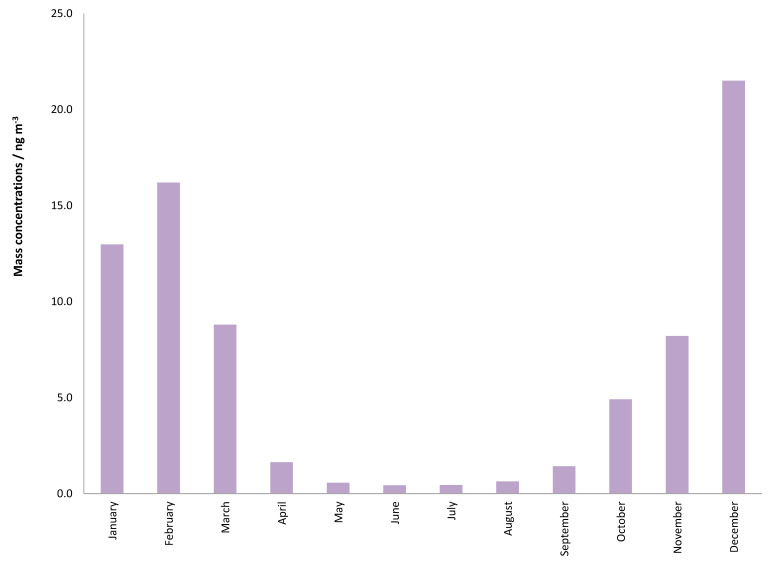
Monthly mass concentrations of ƩPAHs (polycyclic aromatic hydrocarbons) measured during one calendar year.

**Figure 3:**
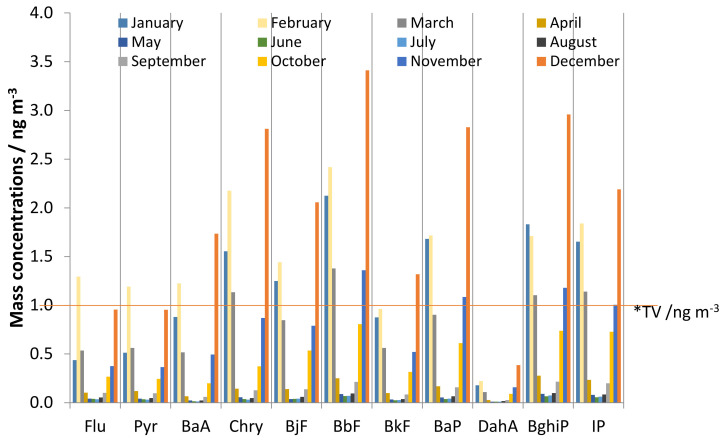
Monthly mass concentrations of PAHs measured during one calendar year. * The target value (TV) in the European Union set by Directive 2004/107/EC for BaP content in PM_10_ fraction is 1 ng m^−3^ averaged over a calendar year.

The monthly average mass concentrations of the PM_1_ particle fraction during the whole calendar year are shown in Figure 1. This result shows seasonal differences (*p* < 0.001) of PM_1_ concentrations with high values during the cold period of the year (January–March; October–December) and lower values during the warm period (April–September). The average 24-h concentrations of PM_1_ varied from 0.7 to 55.3 μg m^−3^ with an annual average of 13.6 μg m^−3^. Monthly average concentrations were also calculated for the PAH sum and for individual PAHs, and the results are presented in Figure 2 and Figure 3. Sums of PAHs concentrations (ΣPAHs) ranged from 0.437 ng m^−3^ during June to 21.497 ng m^−3^ during December, and the annual mean mass concentration was 6.354 ng m^−3^. The highest mass concentration during the cold period was measured for BbF, while in the warm period, the highest mass concentrations were determined for BghiP. High BghiP mass concentrations in the warm period in comparison to other hydrocarbons were probably due to BghiP stability at high temperatures. The lowest mass concentrations for both periods (cold and warm) were determined for DahA. The range of the monthly average concentrations for DahA was from 0.010 ng m^−3^ (May) to 0.386 ng m^−3^ (December). The average monthly mass concentrations of BaP ranged from 0.038 ng m^−3^ (June) to 2.826 ng m^−3^ (December), while the annual mass concentrations were 0.765 ng m^−3^. The target value in the European Union set by Directive 2004/107/EC for BaP content in the PM_10_ fraction is only 1 ng m^−3^ averaged over a calendar year. The reason for higher concentration exclusively during the cold part of the year can be explained because of the long temperature inversion periods that occur in Zagreb. Such weather conditions are characterized by high pressure and impaired air mixing, which favors the pollutants accumulation. These stable atmospheric conditions combine with increased emissions from heating are the basic origins of elevated concentrations of PAHs during the cold season. In the study area, gas, oil or, wood are mostly used for domestic heating, while coal has not been in use for more than thirty years.

The relevant literature comprises a very limited number of papers related to PAHs in the PM_1_ fractions [19,20,21,22,23,24,27,28,40,41]. Table S2 of the Supplementary Data section shows the summary results of PAH mass concentrations obtained in this study in comparison with similar studies. Rogula-Kozłowska et al. [40], Kozielska et al. [22], and Majewski et al. [20] reported much higher mass concentrations of PAHs in the PM_1_ particle fraction during both the heating and non-heating seasons at different locations in Poland compared with Zagreb. Much higher winter concentrations were also found in Ostrava-Radvanice (industrial site), in Kladno-Švermov and Brno (urban sites) in Czech Republic, while levels of PM_1_ and ΣPAHs were similar in Košetice and Čelakovice. In a coastal area in Poland (Baltic Sea), BaP levels were much higher than in this study, while in a coastal area in Greece PM_1_ the levels were similar as in Zagreb but particle-bound PAHs were significantly lower. Similar PAH concentrations were also found in Guadalajara Metropolitan Area in Mexico while PAH concentrations determined in the Metropolitan Area of Porto Alegre, Brazil, were much lower during winter but higher during summer [28].

As significant differences were found between cold and warm months, we divided the results into two groups, separately for the cold (January–March; October–December) and the warm (April–September) period of the year. Summary statistical parameters for all of the measured PAHs for these two periods are shown in Table 1. Concentrations of PAH characteristic for domestic heating or biomass burning (Flu, Pyr) were lower in the cold period than those of PAHs characteristic for car exhausts (BghiP, BbF, and IP). This indicated that the PAHs in the PM_1_ particle fraction could originate predominantly from car exhausts [42,43,44].

**Figure 4:**
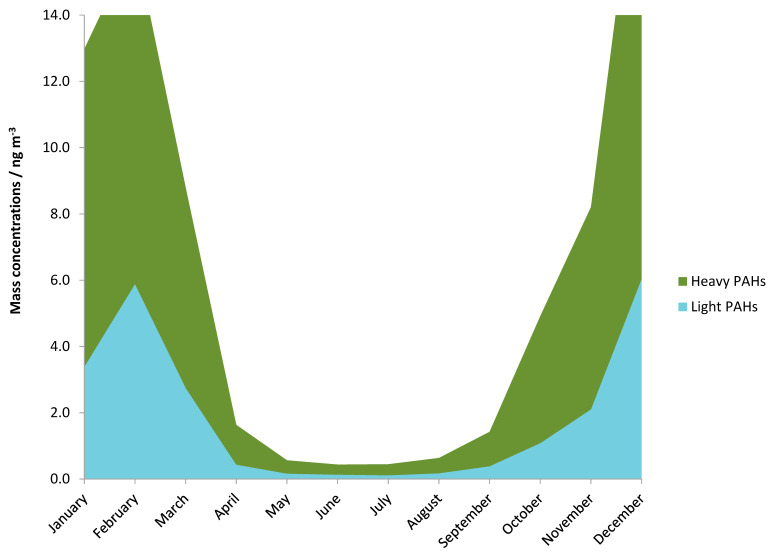
Mass concentrations of light and heavy PAHs measured during one calendar year.

According to the number of rings and molecular weight, PAHs can also be classified into two groups: heavy and light. Heavy PAH concentrations were calculated as the sum of PAHs with five or more aromatic rings, and light PAHs represent the sum of PAHs with four aromatic rings. Heavy PAHs are usually characteristic for car exhausts, while light PAHs originate mostly from domestic heating or biomass burning [43,44] (Figure 4). Figure 4 shows the monthly mass concentration for these two groups. During both measurement periods, the contributions of heavy PAHs were much higher (>69%) than those of light PAHs (Table 2). Concentrations of PM_1_-bounded PAHs during warm periods can easily evaporate from particles to gas phase, so their concentrations in the gas phase increased, but in the particle phase their concentrations decreased. The reason behind this could be that for these PAHs the dominant sources were biomass burning and during the warm period the effect of these sources were minimal. These results also indicated that traffic (diesel or gasoline) could be the main pollution source of PAHs in PM_1_ in this area. To confirm the assumption, we performed diagnostic ratio and principal component analysis to identify possible pollution sources.

### 3.2. Diagnostic Ratios

Specific ratios of individual PAHs are characteristic for some combustion processes and many authors used diagnostic ratios of PAHs to identify potential pollution sources [10,11,12,13]. In this study, the following ratios were selected: IP/(IP + BghiP), BaP/BghiP, Flu/(Flu + Pyr), and BaP/(BaP + Chry). The values determined in this study were compared with the same diagnostic ratios computed from characteristic emission sources based on the relevant literature. IP/(IP + BghiP) ratio values between 0.35 and 0.7 are characteristic for diesel, while some authors reported a value of 0.56 that indicates coal combustion [28,45]. BaP/BghiP values between 0.3 and 0.4 are characteristic for traffic, 0.46–0.81 for diesel combustion, and 0.9–6.6 for coal combustion [28,31,46]. A Flu/(Flu + Pyr) ratio between 0.2 and 0.5 indicates diesel, a ratio between 0.4 and 0.5 liquid fossil fuel, while a ratio >0.5 suggests wood combustion [32,33]. Finally, a BaP/(BaP + Chry) ratio of <0.5 indicates diesel but >0.5 gasoline [4,28]. Average PAH diagnostic ratios for the cold and warm period are presented in Table 3.

**Figure 5:**
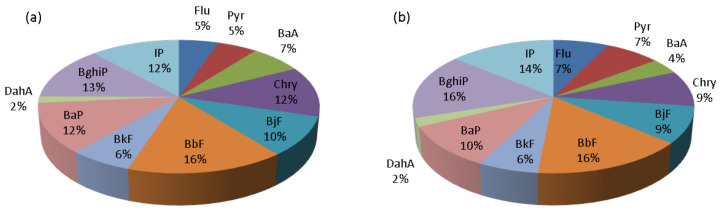
The contributions of the individual PAHs to the sum of total PAH mass concentration during: (**a**) cold and (**b**) warm period.

In this paper, the average IP/(IP + BghiP) ratios were 0.5 during both the cold and warm periods, suggesting that the produced PAHs stemmed from the emission of diesel vehicles. Those average values (0.5) for cold and warm period also corresponded to the 97th and 99th percentile value, respectively, i.e., during the cold period 97% of days had a ratio value lower than 0.5, while during the warm period 99% days were with a ratio of <0.5. The average BaP/(BaP + Chry) and Flu/(Flu + Pyr) ratios were 0.5 during both periods. The average ratio BaP/(BaP + Chry) in the same time represents the 60- and 67-percentile for cold and warm periods, respectively. The other ~40% of results were higher than average value, and the individual value did not exceed 0.7, indicating both diesel and gasoline combustion as potential sources. The average ratio value Flu/(Flu + Pyr) was marginal between biomass burning, emissions from gasoline or diesel vehicles, and combustion of other liquid fossil fuels, but during the warm period there were more days (15%) with a ratio value characteristic for wood burning (during the cold period 96% of results was <0.5). Because of that, it could be concluded that during the warm period mixed sources are probably present (wood combustion and emissions from gasoline or diesel vehicles). The BaP/BghiP ratio was 0.9 during the cold and 0.6 during the warm period. In the warm period, the value was characteristic for emission from diesel (Table 3), but in the cold period it was characteristic for mixed sources (coal, wood combustion, and emission from diesel). The BaP/BghiP ratio included 70% and 60% results during the cold and warm period, respectively. The other 30% during the cold period were higher than 0.9, which indicated that coal or wood combustion were present as pollution sources. During the warm period, 40% of results were higher than 0.6 but did not exceed 0.8, which is the highest value for diesel combustion. Similar results were reported by Agudelo-Castañeda and Teixeira [28] and Hanedar et al. [46]. They also found that PAHs in PM_1_ originated predominately from diesel or gasoline emission. The contributions of the individual PAHs to the sum of total PAH mass concentration were calculated as well and are presented in Figure 5.

From Figure 5 it is evident that the PAH mixture was characterized by a high contribution of 6-ring (BghiP, IP) and 5-ring PAHs (BaP, BjF, BbF, BkF) characteristic for vehicle exhaust emission. Contributions of Flu and Pyr (markers for biomass burning) in the cold period were similar to warm period and it was 5% in cold and 7% in warm period. Previous investigations at the same measuring site but in the PM_10_ particle fractions showed similar results during heating season [26]. These results suggest that four-ring PAHs concentrations have similar dominant sources during the warm and cold period (probably emission from gas and vehicle exhaust and domestic heating). A higher result than those measured in this study was found in Sarajevo [47] and in Poland [40]. Results in Sarajevo and Poland showed a higher contribution of four–ring PAHs (Flu, Pyr, and BaA), which were emitted from coal and wood combustion. The principal compound was BbF (16%) followed by BghiP (13–16%), IP (12–14%), and Chry (9–12%) in both periods. All of them were of pyrolytic origin, which suggests that PAHs reacted at similar extents and the dominant sources were similar.

However, Katsoyiannis et al. [34] presented that the diagnostic ratio cannot be effective as markers of pollution sources, because they can be influenced by atmospheric reaction of PAHs and weather conditions. Because of the fact that the diagnostic ratio can lead to mistakes in estimating the possible pollution sources, in this paper, PCA analysis was used in order to determine the most probable main pollution sources.

### 3.3. Principal Component Analysis

**Figure 6:**
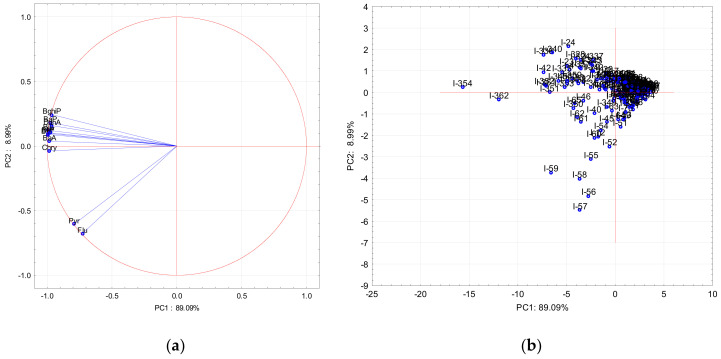
PC1-PC2 loading (**a**) and score plot (**b**) during cold period. I-1 to I-365 are the numbers of days.

Furthermore, for investigating the similarities and differences between samples, PCA was applied, both for the cold and warm period. In the cold and warm period, the eigenvalues of the first two PCs were larger than 1, which indicated their significance. In the cold period, the first two principal components represented 98.08 cum.%, with the first and the second PCs contributing to the total variance of the data set with 89.09 cum.% and 8.99 cum.%, respectively. Results of the PCA during the cold period are presented on the PC1-PC2 loading (Figure 6a) and score plots (Figure 6b), illustrating the orientation of the variables and samples with respect to these principal components.

**Figure 7:**
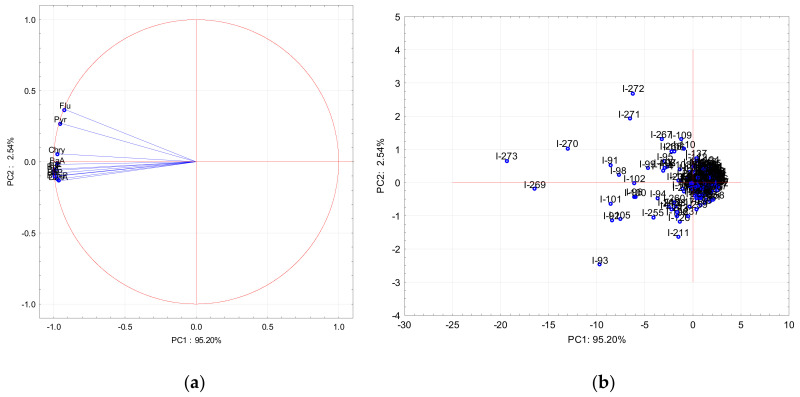
PC1-PC2 loading (**a**) and score plot (**b**) during warm period. I-1 to I-365 are the numbers of days.

All variables had a negative effect on PC1, while all PAHs had positive values on PC2 except for Flu, Pyr, and Chry. Chry showed the lowest negative values. The highest negative effect on PC2 was for Pyr and Flu. In the warm period the first two principal components represent 97.74 cum.% whereas the first and the second PCs contributed with 95.20 cum.% and 2.54 cum.%, respectively, to the total variance of the data set. Results of the PCA during the warm period are presented on the PC1-PC2 loading (Figure 7a) and score plots (Figure 7b), illustrating the orientation of the variables and samples with respect to these principal components.

All of the variables also had a negative effect on PC1 as in the cold period, while Flu, Pyr, and Chry exhibited positive values on PC2, but the rest of PAHs showed negative values. Based on these parameters, two different factors were extracted during both periods. In the cold period, Flu and Pyr were grouped separately because their common origin was different from the rest of the PAHs. These are PAHs characteristic for biomass burning, and their sources probably could have been domestic heating [4,28,48,49]. In the warm period, these two PAHs (Flu and Pyr) were extracted separately probably because they evaporated easily from particles, which makes them more sensitive to meteorological conditions than the other PAHs [50]. However, the results obtained by PCA analysis confirmed those obtained by the diagnostic ratio, but with limitations in the warm period when processes such as meteorological condition and evaporation come into play. PCA analysis secluded some samples during the warm and cold period. From the PC1-PC2 loading, it is evident that six samples during the warm period (Figure 7b) and seven samples during the cold period (Figure 6b) were secluded from other samples. Results of contribution of the individual PAHs to the sum of total PAH for only those selected days showed some differences from the contribution made for all measured warm and cold periods. During warm period samples, I-271 and I-272 had the highest contribution of Flu and Pyr, but samples I-93, I-269, I-270, and I-273 had the highest contribution of BaP except for the I-273 sample, which also had the highest contribution of Chry. During the cold period, samples I-55-59 had the highest contribution of Flu and Pyr, and samples I-354 and I-362 had the highest contributions of BaP, Chry, and BaA. At the end of February and beginning of March (samples I-58 to I-59), the air temperature was extremely lower (from -2 to -10 °C) and probably emission from domestic heating was higher, which caused a higher contribution of Flu and Pyr to the total PAH concentration. For days at the end of December (I-354 and I-362), the air temperature was not as extremely low as in February but nevertheless below zero, and these days are also celebratory days, which can cause high concentrations of PAHs. For the warm period, at the end of September (samples I-269 to I-273) the air temperature was approximately 10 °C, which is much lower than the average value for the warm period (20 °C). Results of PCA analysis were in very good agreement with diagnostic ratios, and for those days, the diagnostic ratios showed mixed sources (diesel/gasoline and wood combustion).

PCA analysis extracted two factors during the cold and warm periods: the first factor separated Flu and Pyr, and the second factor the rest of the PAHs. Because of that, wind roses were shown for two groups, the sum of Flu and Pyr concentrations (ƩFlu+Pyr) and the sum of the remaining nine PAHs (ƩRest PAHs). The dependence of PAH concentrations on wind direction is shown on Figure S1 of the Supplementary Data section together with wind frequencies and wind velocities.

Winds coming from ENE were the most frequent during the cold and warm periods, followed by winds from NNE and SE-ESE sector, while the winds from other directions were relatively rare. On the other hand, winds of high velocities came from north-western and south-western directions, as well as from NE-ENE and SE sections. In the cold months, the highest concentrations of ƩFlu+Pyr primarily came from the west (residential part with domestic heating and street with modest density) and then from the south (center of the town, with dense traffic, while in the warm months the highest concentrations came from the east (industrial part of the town). The difference in concentrations in the cold and warm part of the year is greater than 80%. For the ƩRest PAH wind roses look similar as for ƩFlu+Py, but in the cold months of the year the dominant direction was SSE and then W, while in the warm months the dominant source of pollution was the east (Figure S2). In the cold periods of the year, pollution concentrations from the east were completely absent, which indicated that in the cold months the dominant source of pollution came mostly from the west for ƩFlu+Pyr while for ƩRest PAH it can be concluded that the dominant source of pollution was located both in the west and in the SSE direction. Taking into account the general absence of winds from west direction it can be concluded that the dominant sources of PAHs are probably of local origin. In the warm months of the year, there was no direction from which there was no pollution.

### 3.4. Carcinogenic Activity of the PAH Mixture and Health Impact

The BaP equivalent concentration and total carcinogenic potency were calculated according to Equation (1) and are shown in Table 4. In the cold period of the year, the TCP was more than ten times higher than in the warm period. The average TCP in the warm period was 0.141 ng m^−3^ and in the cold 2.139 ng m^−3^. These results were similar to the TCPs obtained previously in Zagreb [26]. In a study by Pehnec and Jakovljević [26], TCP values were shown for 30 samples collected per season (spring, summer, autumn, and winter) and TCP values varied from 0.063 ng m^−3^ during summer to 4.503 ng m^−3^ during winter. TCP values similar or lower than the ones in this study were reported by other authors who used the same TEF scheme [20,28], while higher TCP values were reported for some locations in Poland [22,51] and the Czech Republic [24].

Contributions of BaP to the carcinogenic potency in this study exceeded 60%. These values were similar for both measuring periods and were on average 62% and 67% for the warm and cold season, respectively. This indicates that benzo(a)pyrene had an important role in the carcinogenic activity of the PAH mixture. The same contribution was reported by Jakovljevic et al. [29] for the same location. Many authors reported similar BaP contributions but in other (PM_10_ or PM_2.5_) particle fractions [46,52].

To determine the daily population exposures, total carcinogenic potency was used to calculate the daily dose according to Equation (3). In this study, we tried to estimate the most probable scenario for three age groups: infant (0–1 year), children (5–19 year), and adult (20–70 year). If we assumed that people spent an average ten hours on their job/school and eight to ten hours at home (including sleeping), we can assume that they were elsewhere for the rest of their day, due to the fact that people spend approximately 25% of their time outdoors. 

The incremental lifetime cancer risk (ILCR) posed by exposure to PM-bounded PAHs was computed following Equation (2) [20].

We used Equation (3) for calculations of IEL for the warm and cold period, for three age group and the results are shown in Table 5.

As the variables in the ILCR calculation belong to certain distributions taking the average value into account can be misleading for yielding the risk estimation. Therefore, we employed a probabilistic Monte Carlo (MC) simulation to feed the distributions to the ILCR model for infants, children, and adults in cold and warm seasons. All of the sampled variables were sampled randomly and independently. The information on variable distributions was taken from Chen and Lia [17] and Liu et al. [18]. We sampled the TCP from our own data after log-transforming them, separately for warm and cold seasons. The MC was iterated 10,000 times per sample variable and finally for the IEL and ILCR models. The parameter values used for MC are show in Table S3 of Supplementary Data. The average ILCR value and the MC simulation results for ILCR were than compared.

IEL was lower during the warm than cold period; 5.04 ng day^−1^, 21.0 ng day^−1^, and 27.7 ng day^−1^ in the warm period for infants, children, and adults, respectively. In the cold period, the BaPeq daily dose was higher; 76.5 ng day^−1^, 319.2 ng day^−1^, and 420.2 ng day^−1^ for infants, children, and adults, respectively. Exposure time is a parameter that influences the inhalation risk most strongly. The incremental lifetime cancer risk (ILCR) was calculated according to Equation (2) and it was 3.35 × 10^−10^ for infants, 4.56 × 10^−9^ for children, and 1.08 × 10^−8^ for adults in the cold period, but in the warm period the ILCR was 15 times lower than in the cold. According to US EPA [53], a one in a million chance of developing an additional human cancer over a lifetime (ILCR = 10^−6^) is considered to be an acceptable level risk. A lifetime risk of one in a thousand (ILCR = 10^−3^) was considered to be a serious health threat. The results determined in this study were much lower than the acceptable risk level of 10^−6^. Higher ILCR values were found by Majewski et al. [20], who calculated the ILCR for students and lecturers at Gliwice and Warsaw University. They found that the ILCR of students exposed to PAHs bounded to the PM_1_ particle ranged from 5.49 × 10^−8^ (Warsaw) to 1.43 × 10^−7^ (Gliwice). However, these results were also below the acceptable risk level of 1 × 10^−6^.

### 3.5. Influence of Meteorological Conditions and Gaseous Pollutants on PAH Concentrations

Spearman’s correlation matrix (Table 6 and Table 7) showed that most of the PAHs measured in the cold and warm periods correlated well with each other (*p* < 0.05). The correlation between PAHs was very strong and coefficients ranged from 0.52 to 0.99 in the cold and from 0.81 to 0.99 in the warm period. These very high correlation coefficients suggested their shared sources and activities. Negative correlations were established between all of the PAHs and temperature and between PAHs and rainfall in both measuring periods (cold and warm). Humidity and pressure were negatively correlated with concentrations of PAHs in the warm period. During the cold part of the year, long temperature inversion periods may occur. Such weather conditions are characterized by high pressure and impaired air mixing, which favors pollutant accumulation. These stable atmospheric conditions combined with increased emissions are the basic origins of elevated concentrations of PAHs during the cold season.

The negative correlation with temperature can be explained with the ability of PAHs to easily vaporize from particle to gas phase [50,54]. A significant correlation was found in the cold period between CO, SO_2_, and all PAHs, as was a positive correlation between NO_2_ and PAHs with high molecular weight, while a negative weak correlation but still statistically significant was established between heavy PAHs and O_3_. Similar correlations were found during the warm period except between PAH and SO_2_, where the correlation was not significant. As there is no industry at the location, SO_2_ was mostly emitted from vehicles [55]. Previous studies have shown that SO_2_ concentrations have been continuously decreasing over the last ten years, the reason for which is the fact that coal has not been in use for more than thirty years at this location [56]. Positive correlations between PAHs and NO_2_ were in good agreement with other studies [55,57]. Previous studies have shown that NO_2_ is one of the main traffic-related air pollutants, and in reaction with PAHs, it can produce nitro PAHs in the atmosphere [55]. In both measuring periods, a negative weak correlation but still significant was found between ozone and PAHs. Ozone is a reactive atmospheric pollutant, and it is able to, in the presence of heat and sunlight, react with PAHs so this was probably one of the reasons for the low PAHs concentrations during summer [55,58]. Positive correlations between CO and PAH suggested their shared sources and activities in both the cold and warm period. Similar results were found in other studies [59].

## 4. Conclusions

Measurements of PAHs in the PM_1_ particle fraction at an urban location in Zagreb, Croatia, showed seasonal differences with higher mass concentrations of PAH in the cold than in the warm period. The annual mass concentration for BaP was 0.765 ng m^−3^, which indicated that the value set by Directive 2004/107/EC for BaP of 1 ng m^−3^ has not been exceeded. During both measurement periods, the contributions of heavy PAHs, characteristic for vehicle emissions, were much higher than those of light PAHs. Results of diagnostic ratios and PCA showed that the main emission source of PAHs associated with PM_1_ in this study area was engine combustion of diesel or gasoline during the warm period but that in the cold period emission from domestic heating is, in addition to diesel, the dominant source. The total carcinogenic potency was estimated using toxic equivalency factors. The average TCP was 0.14 ng m^−3^ and 2.14 ng m^−3^ for the warm and cold period, respectively. The incremental lifetime cancer risk (ILCR) was determined for three age groups of the population: infants (0–1 year), children (5–19 year), and adults (20–70 year) and was below the maximum acceptable level (1 × 10^−6^), revealing that carcinogenic risk posed to the three age groups via inhalation is acceptable. However, more than ten times higher PAH values in the cold part of the year, as well as associated health risk, emphasize the need for regular monitoring of PAH levels in smaller particle fractions, such as PM_1_. These data are a valuable tool in future plans and actions to control PAH sources and to improve the quality of life of urban populations.

## Figures and Tables

**Table 1:** Average, minimum, and maximum values and standard deviations of the PAH 24-h mass concentrations during the cold and warm period (ng m^−3^).

	Warm				Cold			
	C_min_	C_max_	C	SD	C_min_	C_max_	C	SD
*N* = 182					*N* = 181			
Flu	0.009	0.362	0.063 *	0.051	0.041	3.718	0.631 *	0.622
Pyr	0.007	0.358	0.062 *	0.056	0.040	2.896	0.627 *	0.560
BaA	0.008	0.302	0.033 *	0.038	0.051	5.535	0.828 *	0.829
Chry	0.016	0.619	0.074 *	0.078	0.106	8.338	1.464 *	1.320
BjF	0.010	0.579	0.076 *	0.082	0.123	5.209	1.141 *	0.845
BbF	0.027	0.852	0.131 *	0.125	0.228	8.600	1.894 *	1.389
BkF	0.010	0.340	0.052 *	0.051	0.089	3.185	0.751 *	0.539
BaP	0.011	0.665	0.088 *	0.098	0.150	7.483	1.454 *	1.216
DahA	0.004	0.087	0.017 *	0.013	0.021	1.003	0.189 *	0.155
BghiP	0.022	0.792	0.137 *	0.134	0.235	7.027	1.556 *	1.116
IP	0.019	0.748	0.120 *	0.120	0.231	5.250	1.413 *	0.897
ƩPAH	0.177	5.705	0.852 *	0.828	1.384	57.146	11.815 *	9.082

C_min_—minimum value; C_max_—maximum value; C—arithmetic mean; SD—standard deviation; and N—number of samples. * Seasonal differences were tested by Man–Whitney U test (*p* < 0.001).

**Table 2:** Contribution of light and heavy PAHs in the sum of measured PAHs during cold period and warm period.

Parameter	Light PAH		Heavy PAH	
	Cold	Warm	Cold	Warm
Average/ng m^−3^	3.516 *	0.232	8.308 *	0.620
Contribution/%	29.4	27.2	69.5	72.8

* Seasonal differences between warm and cold period (test by Mann–Witney U test; *p* < 0.001).

**Table 3:** Comparison of PAH average diagnostic ratios in PM_1_ during cold and warm period and the main emission sources.

Diagnostic Ratio	Literature Data			This Study		
	Ratio Value	Source	Reference	Average	Percentile	Potential Source
Flu/(Flu + Pyr)	0.2–0.5 0.4–0.5 >0.5	diesel liquid fossil fuel wood combustion	[4] [33] [32]	Cold 0.5 Warm 0.5	96 85	diesel, liquid fossil fuels
BaP/BghiP	0.3–0.4 0.46–0.81 0.9–6.6	traffic diesel coal or wood combustion	[28] [46] [31]	Cold 0.9 Warm 0.6	60 67	diesel/wood diesel
BaP/(BaP + Chry)	<0.5 >0.5	diesel gasoline	[4] [28]	Cold 0.5 Warm 0.5	70 60	diesel/gasoline
IP/(IP + BghiP)	0.35–0.70 0.56	diesel coal	[28]	Cold 0.5 Warm 0.5	97 99	diesel

**Table 4:** Equivalent BaP_eq_ (ng m^−3^) concentrations for individual PAHs measured during the warm and cold period.

PAH	TEF ^(a)^	BaP_eq_	
		Warm	Cold
Flu	0.001	0.0001	0.001
Pyr	0.001	0.0001	0.001
BaA	0.1	0.003	0.082
Chry	0.01	0.001	0.014
BjF	-	-	-
BbF	0.1	0.013	0.187
BkF	0.1	0.005	0.074
BaP	1	0.088	1.438
DahA	1	0.017	0.187
BghiP	0.01	0.001	0.015
IP	0.1	0.012	0.140
TCP ^(b)^		0.141	2.139
% BaP		62.2	67.2

^(a)^ according to Nisbet and LaGoy (1992) ^(b)^ total carcinogenic potency.

**Table 5:** The BaPeq daily dose (IEL) and the incremental lifetime cancer risk (ILCR) for three age groups.

Parameter	Infant		Children		Adult	
	Warm	Cold	Warm	Cold	Warm	Cold
Age	0–1		5–19		20–70	
IEL/ng day^−1^	5.04	76.5	21.0	319.2	27.7	420.2
ILCR	2.21 × 10^−11^	3.35 × 10^−10^	3.01 × 10^−10^	4.56 × 10^−9^	7.13 × 10^−10^	1.08 × 10^−8^

**Table 6:** Spearman’s correlation coefficients between PAHs, gaseous pollutants, and some meteorological parameters during the cold period.

	Pyr	BaA	Chry	BjF	BbF	BkF	BaP	DahA	BghiP	IP	CO	SO_2_	NO_2_	O_3_	t	RH	P	Rainfall
Flu	0.985	0.648	0.712	0.619	0.656	0.651	0.556	0.564	0.515	0.637	0.425	0.473	0.106	0.227	−0.706	0.3	0.09	−0.167
Pyr		0.718	0.776	0.685	0.721	0.719	0.627	0.624	0.591	0.705	0.472	0.458	0.158	0.178	−0.703	0.32	0.089	−0.164
BaA			0.972	0.965	0.966	0.964	0.963	0.944	0.947	0.932	0.663	0.283	0.455	−0.265	−0.499	0.427	0.097	−0.114
Chry				0.968	0.971	0.971	0.924	0.889	0.918	0.956	0.723	0.411	0.5	−0.138	−0.598	0.427	0.043	−0.142
BjF					0.997	0.997	0.979	0.953	0.981	0.986	0.744	0.384	0.519	−0.269	−0.487	0.444	0.061	−0.139
BbF						0.998	0.979	0.956	0.978	0.985	0.735	0.396	0.478	−0.261	−0.511	0.453	0.091	−0.145
BkF							0.973	0.944	0.973	0.99	0.737	0.388	0.496	−0.253	−0.519	0.446	0.065	−0.145
BaP								0.985	0.99	0.949	0.682	0.304	0.442	−0.374	−0.393	0.444	0.121	−0.113
DahA									0.972	0.908	0.645	0.299	0.379	−0.391	−0.375	0.446	0.159	−0.111
BghiP										0.955	0.712	0.292	0.497	−0.389	−0.375	0.431	0.101	−0.122
IP											0.747	0.409	0.515	−0.216	−0.507	0.444	0.037	−0.145
CO												0.392	0.694	−0.326	−0.406	0.459	−0.035	−0.001
SO_2_													0.178	0.265	−0.518	0.179	−0.012	−0.092
NO_2_														−0.25	−0.023	0.098	−0.226	0.016
O_3_															−0.289	−0.343	−0.322	−0.137
t																−0.275	−0.035	0.199
RH																	0.035	0.286
P																		−0.239

t—temperature, RH—relative humidity, and P—atmospheric pressure. Statistically significant correlation coefficients (*p* < 0.05) are underlined.

**Table 7:** Spearman’s correlation coefficients between PAHs, gaseous pollutants, and some meteorological parameters during the warm period.

	Pyr	BaA	Chry	BjF	BbF	BkF	BaP	DahA	BghiP	IP	CO	SO_2_	NO_2_	O_3_	t	RH	P	Rainfall
Flu	0.97	0.861	0.892	0.887	0.9	0.888	0.877	0.812	0.88	0.878	0.489	0.102	0.448	−0.396	−0.554	−0.013	−0.094	−0.227
Pyr		0.947	0.953	0.92	0.938	0.931	0.93	0.825	0.904	0.912	0.459	0.075	0.382	−0.328	−0.602	−0.072	−0.153	−0.214
BaA			0.983	0.928	0.934	0.938	0.955	0.844	0.895	0.913	0.457	0.109	0.277	−0.28	−0.634	−0.053	−0.227	−0.098
Chry				0.925	0.947	0.946	0.949	0.833	0.914	0.926	0.481	0.115	0.346	−0.325	−0.656	−0.026	−0.239	−0.059
BjF					0.985	0.988	0.989	0.966	0.979	0.983	0.525	0.12	0.337	−0.303	−0.56	−0.0004	−0.202	−0.12
BbF						0.999	0.986	0.927	0.989	0.991	0.531	0.123	0.376	−0.309	−0.578	−0.032	−0.226	−0.143
BkF							0.99	0.93	0.987	0.99	0.52	0.118	0.355	−0.297	−0.58	−0.025	−0.233	−0.131
BaP								0.934	0.971	0.977	0.499	0.115	0.306	−0.286	−0.598	−0.008	−0.215	−0.124
DahA									0.937	0.941	0.575	0.205	0.313	−0.338	−0.483	0.042	−0.151	−0.057
BghiP										0.996	0.535	0.13	0.371	−0.331	−0.573	−0.008	−0.215	−0.123
IP											0.548	0.135	0.367	−0.329	−0.592	−0.008	−0.207	−0.112
CO												0.719	0.513	−0.578	−0.262	0.221	−0.048	0.004
SO_2_													0.023	−0.226	0.001	0.085	−0.052	0.04
NO_2_														−0.613	−0.159	0.222	0.013	−0.029
O_3_															0.45	−0.611	−0.159	−0.118
t																−0.331	−0.163	0.064
RH																	0.262	0.214
P																		−0.161

t—temperature, RH—relative humidity, and p—atmospheric pressure. Statistically significant correlation coefficients (*p* < 0.05) are underlined.

## Supplementary Materials

The following are available online at https://www.mdpi.com/1660-4601/17/24/9587/s1, Table S1: Detection (DL) and quantification limits (QL) and recoveries (R) of PAHs analyzed by high-performance liquid chromatography (HPLC) with a fluorescence detector, Table S2: Comparison of PAH mass concentrations in PM1 particle fraction obtained in this study with other studies, Table S3: Risk parameters for different age groups, Figure S1: Wind roses. Dependence of PAH concentrations (ng m^−3^) on wind direction, average wind velocities (m s^−1^) and wind direction frequencies (%) for cold and warm measuring periods, Figure S2: Distribution of incremental lifetime cancer risk for adults, children and infants, derived using Monte Carlo simulation in cold and warm period.
