# Supplementary material for: Pollution Sources and Carcinogenic Risk of PAHs in PM1 Particle Fraction in an Urban Area
Source: Int J Environ Res Public Health. 2020 Dec 21;17(24):9587. doi: 10.3390/ijerph17249587 (PMC7767419; doi:10.3390/ijerph17249587)
Supplement: Supplementary file 1 [file ijerph-17-09587-s001.pdf]

Table 1. Detection (DL) and quantification limits (QL) and recoveries (R) of PAHs analyzed by high-performance liquid chromatography (HPLC) with a fluorescence detector.

	Flu	Pyr	BaA	Chry	BjF	BbF	BkF	BaP	DahA	BghiP	IP
**DL* (ng m^{-3})**	0.018	0.003	0.001	0.001	0.03	0.01	0.001	0.001	0.01	0.015	0.015
**QL** (ng m^{-3})**	0.06	0.012	0.002	0.002	0.108	0.033	0.004	0.003	0.037	0.050	0.048
**R*** (%)**	88.3	90.2	101.5	104.1	108.3	96.3	109.2	100.0	89.8	106.7	101.6

* the detection limits were determined as concentration equivalents to three times the signal-to-noise ratio. ** the quantification limits were determined as concentration equivalents to ten times the signal-to-noise ratio. *** the accuracy of the method was determined by analyzing a standard reference material (SRM urban dust 1649b) and expressed as percentage recovery

Table S2. Comparison of PAH mass concentrations in PM_1 particle fraction obtained in this study with other studies.

City, Country	Site description	Sampling period	Average PAH (ng m^{-3})	Average PM_1 (µg m^{-3})	Reference
Zagreb, Croatia	urban background	January–December 2018	Warm season: Flu 0.063; Pyr 0.062; BaA 0.033; Chry 0.074; BjF 0.076; BbF 0.131; BkF 0.052; BaP 0.088; DahA 0.017; BghiP 0.137; IP 0.120; ΣPAH 0.852<br>Cold season: Flu 0.631; Pyr 0.627; BaA 0.828; Chry 1.464; BjF 1.141; BbF 1.894; BkF 0.751; BaP 1.454; DahA 0.189; BghiP 1.556; IP 1.413; ΣPAH 11.815	Warm: 8.7<br>Cold: 18.6<br>Annual: 13.6	This study
Zagreb, Croatia	urban background	January 1–February 22, 2014<br>March 22–May 11, 2014<br>June 24–August 7, 2014<br>September 27–November 9, 2014	Winter: Flu 1.214; Pyr 1.210; BaA 0.873; Chry 1.825; BbF 2.293; BkF 1.113; BaP 2.228; DahA 0.317; BghiP 4.199; IP 2.030; ΣPAH 17.274<br>Spring: Flu 0.219; Pyr 0.151; BaA 0.089; Chry 0.176; BbF 0.332; BkF 0.127; BaP 0.205; DahA 0.046; BghiP 0.650; IP 0.268; ΣPAH 2.262<br>Summer: Flu 0.023; Pyr 0.024; BaA 0.025; Chry 0.032; BbF 0.059; BkF 0.027; BaP 0.030; DahA 0.003; BghiP 0.116; IP 0.031; ΣPAH 0.372<br>Autumn: Flu 0.204; Pyr 0.218; BaA 0.197; Chry 0.295; BbF 0.815; BkF 0.358; BaP 0.593; DahA 0.105; BghiP 1.667; IP 0.616; ΣPAH 5.069		[26]
Zagreb, Croatia	urban background	January–December 2013	Flu 0.595; Pyr 0.532; BaA 0.487; Chry 0.925; BbF 1.034; BkF 0.408; BaP 0.816; DahA 0.083; BghiP 1.607; IP 0.819	17.8	[19]
Warsaw, Poland	urban	April 20–June 1, 2015	Flu 2.70; Pyr 0.17; BaA 0.57; Chry 1.21; BbF 0.36; BkF 0.26; BaP 1.54; DahA 0.08; BghiP 0.03; IP 0.09; Σ_{16}PAH 8.08		[20]
Gliwice, Poland	urban	April 20–June 1, 2015	Flu 1.56; Pyr 0.57; BaA 1.99; Chry 1.57; BbF 1.90; BkF 1.40; BaP 2.72; DahA 0.04; BghiP 0.52; IP 0.67; Σ_{16}PAH 14.85		
Athens Basin, Greece	roadside-industrialized	Selected days in 2008	Flu 0.026; Pyr 0.025; BaA 0.023; Chry 0.069; BbF 0.254; BkF 0.143; BaP 0.044; DahA 0.020; BghiP 0.113; IP 0.168; ΣPAH_{EPA} 0.818	21.8	[21]
Athens Basin, Greece	coastal background	Selected days in 2008	Flu 0.015; Pyr 0.011; BaA 0.020; Chry 0.011; BbF 0.013; BkF 0.008; BaP 0.004; DahA 0.004; BghiP 0.042; IP 0.012; ΣPAH_{EPA} 0.176	16.9	
Katowice, Poland	urban background	August 2, 2009–December 27, 2010	Heating season: Flu 23.36; Pyr 20.30; BaA 17.67; Chry 17.30; BbF 9.95; BkF 10.74; BaP 12.48; DahA 0.27; BghiP 4.95; IP 5.03; Σ_{16}PAH 138.74<br>Non-heating season: Flu 2.01; Pyr 1.87; BaA 7.81; Chry 2.48; BbF 0.76; BkF 1.87; BaP 2.97; DahA 0.24; BghiP 0.05; IP 0.35; ΣPAH_{16} 30.26	Heating: 40.70<br>Non-heating: 20.83	[22]

Złoty Potok, Poland	regional background	August 2, 2009–December 27, 2010	Heating season: Flu 3.42; Pyr 1.86; BaA 2.05; Chry 3.26; BbF 1.55; BkF 1.24; BaP 4.03; IP 0.49; DahA 0.21; BghiP 0.30; Σ_{16}PAH 23.10<br>Non-heating season: Flu 2.65; Pyr 1.64; BaA 1.57; Chry 2.81; BbF 0.80; BkF0.68; BaP 2.46; DahA 0.37; BghiP 0.24; IP 0.38; Σ_{16}PAH 18.57	Heating: 16.37<br>Non-heating: 10.32	
Katowce, Poland	urban traffic	August 2, 2009–December 27, 2010	Heating season: Flu 26.69; Pyr 21.51; BaA 19.02; Chry 19.85; BbF 14.23; BkF 13.80; BaP 14.27; DahA 17.21; BghiP 8.29; IP 5.57; Σ_{16}PAH 186.12<br>Non-heating season: Flu 3.62; Pyr 10.90; BaA 10.41; Chry 3.86; BbF 3.38; BkF 0.97; BaP 4.73; DahA 4.77; BghiP 0.09; IP 0.51; Σ_{16}PAH 56.02	Heating: 41.55<br>Non-heating: 18.40	
Gdynia (Baltic Sea), Poland	urbanized coastal zone	January 1–December 31, 2012	BaP only<br>Heating season 3.7 ± 3.9; Non-heating season 0.2 ± 0.4; Average: 2.6 ± 3.6	Heating: 31.5<br>Non-heating: 25.2.<br>Annual: 27.5	[23]
Czech Republic:			Σ_{15}PAH		[24]
Mladá Boleslav	urban	Winter 2013	15.6	26.0	
Ostrava-Radvanice	industrial	Winter 2014	60.8	29.4	
Čelákovice	urban	Winter 2015	11.7	17.6	
Kladno-Švermov	urban	Winter 2016	25.5	18.8	
Brno	urban	Winter 2017	20.7	34.2	
Košetice	rural	Winter 2017	12.3	24.5	
Treviso, Veneto region, Italy	Urban background	January–June 2017	Flu 0.52; Pyr 0.73; BaA 0.84; Chry 1.73; ΣBbkF 3.56; BaP 0.86; DahA 0.06; BghiP 1.01; IP 0.52		[25]
Guadalajara Metropolitan Area, Mexico	urban (traffic, industry) and urban residential site	April–June 2015	Flu 0.0114; Pyr 0.0171; BaA 1.47; Chry 1.53; BbF 1.62; BkF 0.541; BaP 0.0947; DahA 0.354; BghiP 0.584; IP 0.498; Σ_{16}PAH 7.25		[27]
Metropolitan Area of Porto Alegre, Brazil	roadside/traffic	August 2011–July 2013	ΣPAH 1.32 (summer)<br>ΣPAH 2.02 (winter)	8.60<br>11.62	[28]
Metropolitan Area of Porto Alegre, Brazil	urban road/ traffic	August 2011–July 2013	ΣPAH 1.57 (summer)<br>ΣPAH 3.05 (winter)	13.47<br>17.50	

Table S3. Risk parameters for different age groups.

Definition	Units	Infant	Children	Adults
Inhalation cancer slope factor of BaP (SF_{inh})	kg day mg^{-1}	3.14	3.14	3.14
Exposure frequency (EF)	day year^{-1}	252	252	252
Daily exposure level (ED)	μg g^{-1}	4.8x10^{-4}	8.8x10^{-4}	7.1x10^{-4}
Body weight (BW)	kg	6.79	36.24	59.78
Average time (AT)	day	25550	25550	25550
Inhalation rate (IR)	m^3 day^{-1}	5.96	24.87	32.74

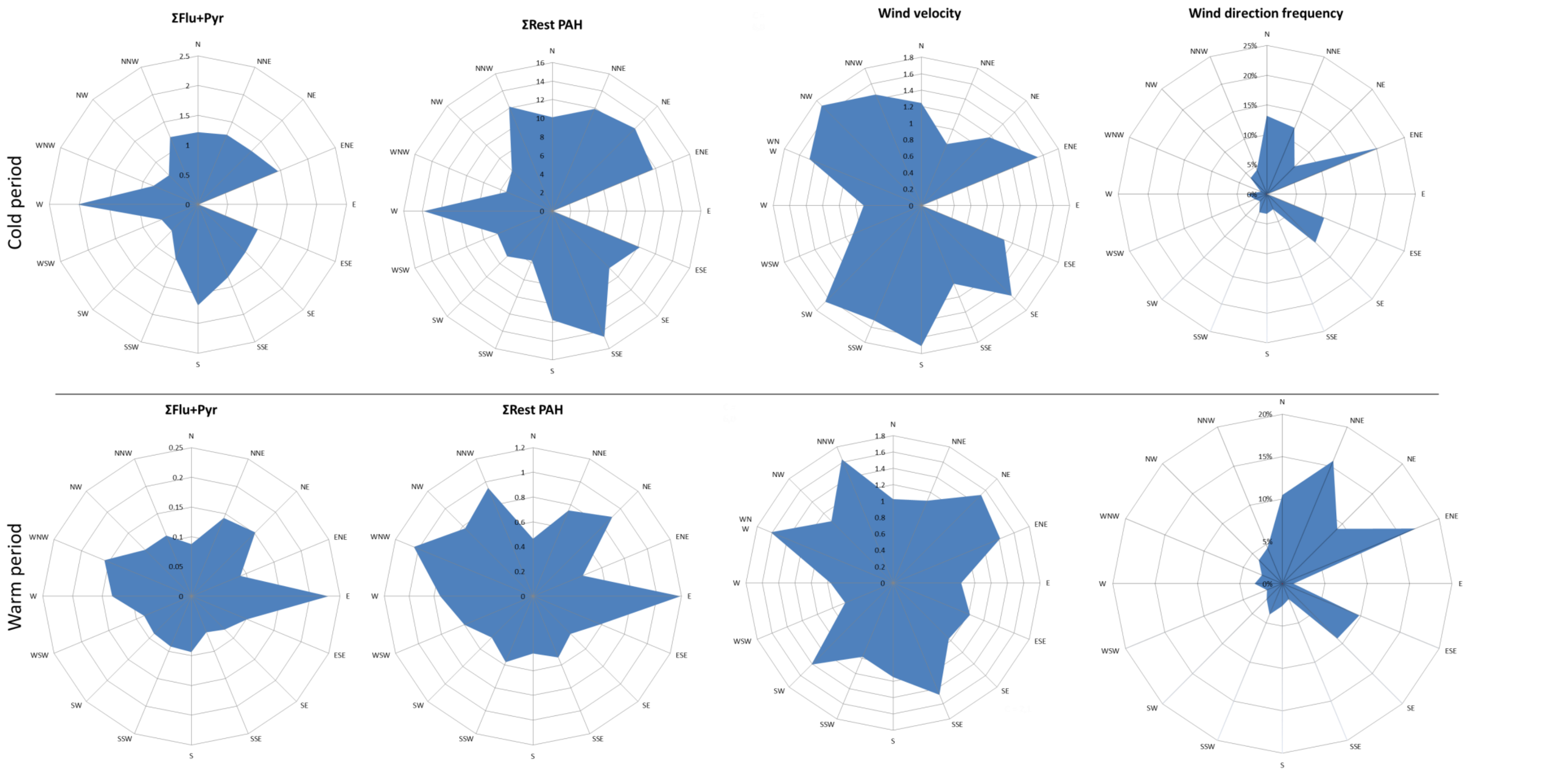

Figure S1. Wind roses. Dependence of PAH concentrations (ng m^{-3}) on wind direction, average wind velocities (m s^{-1}) and wind direction frequencies (%) for cold and warm measuring periods.

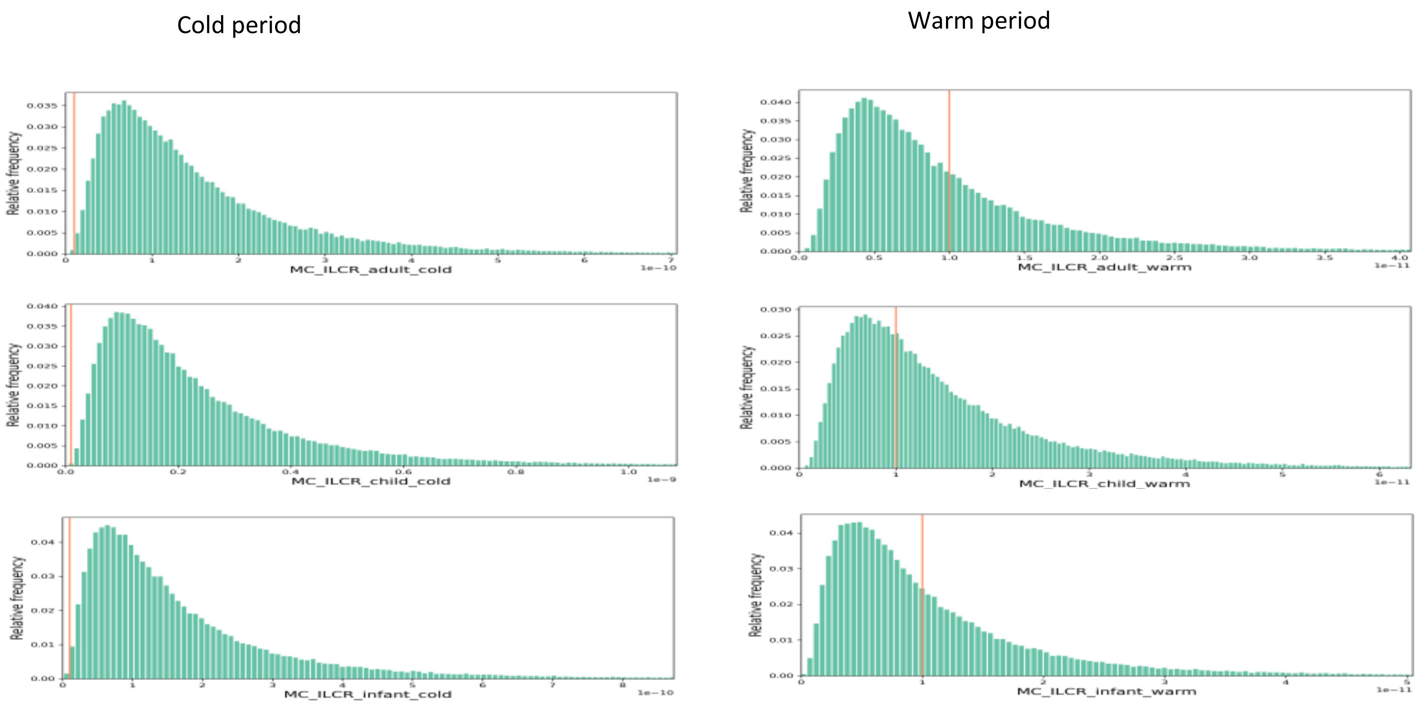

Figure S2. Distribution of incremental lifetime cancer risk for adults, children and infants, derived using Monte Carlo simulation in cold and warm period.
